# *In vivo* functional and molecular characterization of the Penicillin-Binding Protein 4 (DacB) of *Pseudomonas aeruginosa*

**DOI:** 10.1186/s12866-016-0853-x

**Published:** 2016-10-06

**Authors:** Cristian Gustavo Aguilera Rossi, Paulino Gómez-Puertas, Juan Alfonso Ayala Serrano

**Affiliations:** 1 Departamento de Ciencias Preclínicas, Facultad de Medicina, Universidad de La Frontera, Temuco, Chile; 2 Laboratorio de División Celular Bacteriana y Resistencia a Antibióticos, Centro de Biología Molecular “Severo Ochoa”, Universidad Autónoma de Madrid-CSIC, Madrid, Spain; 3 Grupo de Modelado Molecular, Centro de Biología Molecular “Severo Ochoa”, Universidad Autónoma de Madrid-CSIC, Madrid, Spain

**Keywords:** *Pseudomonas aeruginosa*, LMM-PBP4, Purified muropeptides, Macromolecular peptidoglycan, Catalytic function, D,D-peptidase, Three-dimensional structure

## Abstract

**Background:**

Community and nosocomial infections by *Pseudomonas aeruginosa* still create a major therapeutic challenge. The resistance of this opportunist pathogen to β-lactam antibiotics is determined mainly by production of the inactivating enzyme AmpC, a class C cephalosporinase with a regulation system more complex than those found in members of the *Enterobacteriaceae* family. This regulatory system also participates directly in peptidoglycan turnover and recycling. One of the regulatory mechanisms for AmpC expression, recently identified in clinical isolates, is the inactivation of LMM-PBP4 (Low-Molecular-Mass Penicillin-Binding Protein 4), a protein whose catalytic activity on natural substrates has remained uncharacterized until now.

**Results:**

We carried out *in vivo* activity trials for LMM-PBP4 of *Pseudomonas aeruginosa* on macromolecular peptidoglycan of *Escherichia coli* and *Pseudomonas aeruginosa*. The results showed a decrease in the relative quantity of dimeric, trimeric and anhydrous units, and a smaller reduction in monomer disaccharide pentapeptide (M5) levels, validating the occurrence of D,D-carboxypeptidase and D,D-endopeptidase activities. Under conditions of induction for this protein and cefoxitin treatment, the reduction in M5 is not fully efficient, implying that LMM-PBP4 of *Pseudomonas aeruginosa* presents better behaviour as a D,D-endopeptidase. Kinetic evaluation of the direct D,D-peptidase activity of this protein on natural muropeptides M5 and D45 confirmed this bifunctionality and the greater affinity of LMM-PBP4 for its dimeric substrate. A three-dimensional model for the monomeric unit of LMM-PBP4 provided structural information which supports its catalytic performance.

**Conclusions:**

LMM-PBP4 of *Pseudomonas aeruginosa* is a bifunctional enzyme presenting both D,D-carboxypeptidase and D,D-endopeptidase activities; the D,D-endopeptidase function is predominant. Our study provides unprecedented functional and structural information which supports the proposal of this protein as a potential hydrolase-autolysin associated with peptidoglycan maturation and recycling. The fact that mutant PBP4 induces AmpC, may indicate that a putative muropeptide-subunit product of the DD-EPase activity of PBP4 could be a negative regulator of the pathway. This data contributes to understanding of the regulatory aspects of resistance to β-lactam antibiotics in this bacterial model.

**Electronic supplementary material:**

The online version of this article (doi:10.1186/s12866-016-0853-x) contains supplementary material, which is available to authorized users.

## Background

*Pseudomonas aeruginosa* behaves as an opportunist pathogen capable of affecting a wide range of tissues and generating clinical infection episodes which compromise the host’s defence mechanisms [1]. Infections with *Pseudomonas aeruginosa* represent a major therapeutic challenge in which choice of the right antibiotic is fundamental, however this choice is complicated by the fact that *P. aeruginosa* presents natural resistance to antibacterial agents to which it is not structurally related, and can even acquire resistance during treatment [2, 3]. Bacterial resistance to β-lactam antibiotics is determined mainly by the production of inactivating enzymes. AmpC of *P. aeruginosa* is a naturally inducible enzyme. Wild-type strains may be susceptible to anti-*Pseudomonas* penicillin, inhibitor-penicillin combinations, cephalosporins and carbapenems, however in the presence of a β-lactam inducer, an increase in AmpC production may cause resistance to almost all known β-lactams, except carbapenems [4]. AmpC overproduction may occur through reversible induction of *ampC* expression during exposure to certain β-lactams (cephamycins and carbapenems) and β-lactamase inhibitors (clavulanic acid). An important treatment fail occurs when *ampC* regulation is lost due to de-repression, this condition generally involves genetic mutations in proteins responsible for regulating *ampC* expression [4, 5]. In *P. aeruginosa*, *ampC* gene induction is closely connected to peptidoglycan recycling, but regulation of the expression is not yet fully understood [6]. Recycling of peptidoglycan is a highly regulated system, allowing the bacterial cell wall to be efficiently remodelled during growth and division without its integrity being compromised. It has been proposed that *P. aeruginosa* is capable of “sensing” the disturbance of its cell wall, inducing expression of β-lactamase AmpC and recovering homeostasis of the murein by hydrolysis of the antibiotic [7. During normal growth and division, peptidoglycan fragments are eliminated by autolysins to produce a series of periplasmic peptides, GlcNAc-1,6-anhydroMurNAc-peptides (tri, tetra or pentapeptides), transported to the cytoplasm through AmpG and AmpP internal membrane permeases [8]. In the cytoplasm, GlcNAc is eliminated from the muropeptide by the action of NagZ (β-N-acetylglucosaminidase), and the pool of 1,6-anhydroMurNAc-peptides is recycled to form UDP-MurNAc-pentapeptide, a precursor of peptidoglycan which will be reincorporated into the murein of the cell wall. Tripeptide and pentapeptide species have been proposed as effector molecules which induce *ampC* transcription through competitive binding with AmpR (LysR transcriptional regulator) [7, 9]. In the absence of β-lactam antibiotics, the cytoplasm concentration of 1,6-anhydroMurNAc-peptides is controlled by the activity of AmpD, a N-acetylmuramoyl-L-alanine amidase which removes the peptide chain from 1,6-anhydroMurNAc and GlcNAc-1,6-anhydroMurNAc, reduces its concentration and prevents overexpression of AmpC. Thus UDP-MurNAc-pentapeptide predominates and binds to AmpR, favouring repression of *ampC* transcription. Exposure to β-lactams (inductors) stops peptidoglycan synthesis and increases fragmentation, favouring the accumulation of 1,6-anhydroMurNAc-peptides and probably allowing tripeptide or pentapeptide species to displace UDP-MurNAc-pentapeptide from the AmpR regulator, determining the activation of the *ampC* gene [7–9].

Penicillin-Binding Proteins (PBPs) are acyl-serin transferases, initially identified by their ability to form covalent bonds with penicillin [10]. They are located on the external face of the inner membrane and share common D,D-peptidase activities (D,D-transpeptidase, D,D-carboxypeptidase or D,D-endopeptidase), catalysed by a domain which binds β-lactams (PB domain). The active site of these proteins contains well preserved residues with principal sequence S*xxK containing catalytic serin and the SxN and KTG triads, which together allow acetylation and deacetylation processes [11, 12]. PBPs can be classified into two main categories as a function of their amino acid sequence and relative mobility in SDS-PAGE gels: high molecular mass PBPs (HMM-PBPs, >60 kDa) and low molecular mass PBPs (LMM-PBPs, <60 kDa). HMM-PBPs are responsible for the polymerisation of peptidoglycan and its incorporation into the pre-existing cell wall, while LMM-PBPs are involved in peptidoglycan cell separation, maturation and recycling, acting as catalyst for D,D-carboxypeptidase or D,D-endopeptidase activities [13, 14]. Five HMM-PBPs are recognised for the *Pseudomonas aeruginosa* model: PBP1a, PBP1b, PBP2, PBP3 and PBP3a(3x); and three LMM-PBPs: PBP4, PBP5(6) and PBP7, which are homologous with PBP1a, 1b, 2, 3, 4, 5 and 7 of *E. coli*. Only the coding genes for PBP1a, 2, 3, 3a (3x) and 5 have been cloned and characterised [15, 16]; the crystallographic structure has been resolved for HMM-PBP3 [17] and LMM-PBP5 [12].

Modification of any protein involved in the *ampC* induction mechanism may lead to de-repression of the expression of this gene. Although research into members of the *Enterobacteriaceae* family has identified mutations in *ampR*, the majority of the changes observed in clinical isolations of *P. aeruginosa* have been associated with the *ampD* gene [7 9 *,* 18 *]*. The AmpD protein has been characterised as a negative AmpC regulator, however mutations in the coding gene compromise normal processing of anhydromuropeptides, producing a permanent increase in the concentration of cytoplasm effectors which favours binding with AmpR and raises the constitutive expression of *ampC* [2, 7, 19, 20]. A further complexity for *P. aeruginosa* is that some strains which overproduce AmpC do not present mutations in *ampR* and *ampD* genes, or in the *ampR*-*ampC* inter-gene region, and do not show changes in the expression level of *ampD*, indicating the existence of additional factors contributing to *ampC* regulation in this model. Spontaneous inactivation of LMM-PBP4, encoded by the *dacB* gene, has been recognised as a clinically relevant cause of resistance to anti-*Pseudomonas* β-lactam antibiotics through hyperproduction of AmpC, with levels exceeding those produced by *ampD* mutants. Inactivation of this LMM-PBP provokes a highly efficient and complex response in terms of β-lactam resistance, triggering overproduction of chromosomal AmpC β-lactamase and specific activation of the two-components regulatory system CreBC (BlrAB), which plays a role in resistance through an still unknown mechanism [2 7 *,* 21–23]. The connection between the peptidoglycan turnover and recycling processes, and the regulatory pathways for constitutive hyperproduction of chromosomal AmpC β-lactamase, particularly the mechanism related with LMM-PBP4 inactivation, raised the need for the present work, whose aim is to define the *in vivo* functionality of this LMM-PBP of *Pseudomonas aeruginosa*.

## Methods

### Bacterial strains, plasmids, phages and primers

The genotype and relevant phenotype of bacterial strains, plasmids, and phages, and nucleotide sequences of primers used in this study are given in Additional file 1: Tables S1, S2 and S3 [24–27].

### Cloning, expression and purification of LMM-PBP4

Genomic DNA from the reference strain *Pseudomonas aeruginosa* O1 (PAO1) was extracted and purified using the Wizard® Genomic DNA Purification Kit (Promega); the samples were quantified and stored at 4 °C. The *dacB* gene (PA3047, GenBank accession number AAG06435.1) was amplified using Touchdown PCR in a *MiniCycler*™ PTC-150 (MJ Research) and the products recovered, *dacB*-*Nde*I-*Hind*III, *dacB*-*Nde*I-*Hind*IIITC, d*acB*-*Nco*I-*Hind*III (~1.5 Kb) were purified, digested, bound to expression vector pET-28b(+) and transformed into electro-competent cells of *E. coli* BL21(DE3). Positive clones were recovered from transformant colonies on Luria-Bertani (LB) agar plates supplemented with kanamycin 30 μg/ml, and finally proved by sequencing. Transformed *E. coli* BL21(DE3) containing pET-PBP4HNC (PBP4 recombinant protein with carboxyl and amino His-tag terminals), pET-PBP4HN (PBP4 recombinant protein with amino His-tag terminal) and pET-PBP4HC (PBP4 recombinant protein with carboxyl His-tag terminal) were cultured in LB medium supplemented with kanamycin 30 μg/ml to a DO_600_ of 0.4. Induction of protein expression was achieved by addition of 1 mM isopropyl-β-D-thiogalactopyranoside (IPTG) and incubation for 60 min at 37 °C. Cells were then harvested and frozen at −70 °C. To purify the expressed proteins, a portion of the cell paste was thawed and suspended in phosphate buffer saline (PBS) pH 8.0. Cells were disrupted by two passes through a French® Pressure Cell Press (SLM AMINCO) at 20,000 lb/in^2^, and the lysate was centrifuged in a TL-100 (Beckman) ultracentrifuge at 70,000 rpm for 40 min at 4 °C. The resulting pellet was suspended in PBS (pH 8.0) with 1 % Sarkosyl and stirred for 4 h at room temperature. The insoluble material was separated by ultracentrifuging (80,000 rpm for 40 min at 20 °C) and the supernatant (soluble extract) was dialyzed with PBS 1X, Triton™ X-100 0.2 % pH 8.0 (solubilisation buffer) and mixed with imidazole (20 mM final concentration). The dialyzed material was incubated in one volume of nickel resin Ni-NTA Agarose (Qiagen), previously balanced in PBS 1X, Triton™ X-100 0.2 %, imidazole 20 mM pH 8.0 (rinse buffer) for 4 h at room temperature with gentle rotation. Unbound proteins were removed from the resin by washing with rinse buffer three times. Proteins bound to the nickel-resin were eluted stepwise with 3 ml each of 125 mM, 250 mM and 500 mM imidazole in solubilisation buffer. The samples were dialyzed, aliquoted, quantified, concentrated (in units by Amicon® Ultra-4 Centrifugal Filter 30 kDa MWCO, Millipore) and stored at −20 °C. Fractions of each purified recombinant protein identified as PBP4HNC (NC-terminal His · tag), PBP4HN (N-terminal His · tag) and PBP4HC (C-terminal His · tag), were analysed using SDS-PAGE and immuno detection (western blot).

### Cloning and expression of recombinant construct in *Pseudomonas aeruginosa*

For cloning in an expression vector compatible with *Pseudomonas aeruginosa*, the *dacB* coding gene was amplified using defined primers together with genomic DNA previously extracted and purified from reference strain PAO1. The amplification product *dacB*-*Eco*RI-*Hind*III (~1.5 Kb) was cloned in pHERD26T expression plasmid (*Escherichia*-*Pseudomonas* shuttle vector). The pHERD-PBP4 recombinant clone was transformed into electro competent *Pseudomonas aeruginosa* UCBPP-PA14 (PA14WT) cells, previously prepared according to a protocol described by Choi et al. [28]. Positive transformants (PA14WT/pHERD-PBP4 ~ 7.7 Kb) recovered from colonies cultured on unsupplemented LB agar plates were purified and identified by sequencing (dGTP BigDye® Terminator v3.0, Applied Biosystems). Overexpression was carried out with L(+)-arabinose 0.2 % at 37 °C for 60 min.

### Preparation of bacterial envelopes and identification of Penicillin-Binding Proteins

Membranes of *E. coli* BL21(DE3)/pET-PBP4HNC, *E. coli* BL21(DE3)/pET-PBP4HN and *E. coli* BL21(DE3)/pET-PBP4HC, with and without IPTG induction, were prepared from a cell volume equivalent to 100 ml of culture previously centrifuged at 10,500 rpm for 10 min at 4 °C (Avanti™ J-25, Beckman Coulter). The pellet was re-suspended in 3 ml of PBS 1X pH 8.0 and treated in an ultrasonic homogenizer LABSONIC® M, Sartorius (4 cycles of 60 s, amplitude 90 %). The sound-treated solution was ultracentrifuged at 80,000 rpm for 40 min at 4 °C, and the pellet from each sample (membrane fraction) was re-suspended in 200 μl of PBS 1X pH 8.0 and stored at −20 °C. The affinity and identification assays for LMM-PBP4 were based on modifications of the procedures described by Spratt & Pardee [29]. In β-lactam binding assays, membrane extracts (50 μg) were incubated with BOCILLIN™ FL Penicillin 10 μM (Life Technologies) for 30 min at 37 °C; the reaction was stopped by incorporating 10 μl of NuPAGE® LDS Sample Buffer 4X with reducing agent (2-mercaptoethanol). Samples were boiled for 10 min and the insoluble materials were removed by centrifuging. Proteins in the samples were separated by SDS-PAGE (NuPAGE® Novex® 8 % Bis-Tris Midi Gel) and detected directly on the gels on a Typhoon™ 9410 variable-mode imager (Amersham Biosciences) at 488 nm with a 520BP40 emission filter. The images obtained were processed with the ImageQuant™ TL programme v2003.02 (Amersham Biosciences) and the fluorescent signal was measured in a GS-800™ Calibrated Densitometer (Bio-Rad) using the Quantity One® 1-D Analysis programme v4.6.3 (Bio-Rad).

### Preparation of peptidoglycan

The peptidoglycan was prepared using a standard procedure described by Glauner [30]. Cultures in exponential growth phase in LB medium at 37 °C with aeration were harvested by centrifuging for 15 min at 8500 rpm at 4 °C; re-suspended in 4.5 ml of PBS 1X pH 8.0 and slowly mixed with an equal volume of 6 % (wt/vol) boiling SDS with vigorous stirring for 4 h and left overnight with moderate stirring at room temperature. The insoluble fraction (peptidoglycan) was recovered by ultracentrifuging (80,000 rpm for 30 min at 25 °C) and re-suspended in 3 ml of warm Milli-Q water; it was washed repeatedly by re-suspension and ultracentrifuging (80,000 rpm for 20 min at 25 °C) until complete elimination of the SDS present in the sample. The last pellet was suspended in 900 μl of Tris–HCl 10 mM, NaCl 0.06 % pH 7.2 and digested first with 100 μg/ml α-amylase at 37 °C for 90 min and then with 100 μg/ml of preactivated pronase-E at 60 °C for 60 min. The enzymes were inactivated by boiling for 20 min in 1 % (final concentration) SDS. The SDS was removed after washing 3–4 times in Milli-Q water as described above. The purified sacculus was stored in water at 4 °C until use.

### Preparation and separation of muropeptides

Macromolecular peptidoglycan was re-suspended in 500 μl of 50 mM phosphate buffer pH 4.9 and digested with Cellosyl (Hoechst AG) 100 μg/ml final concentration at 37 °C overnight. The enzyme reaction was stopped by boiling the sample for 15 min in a water bath. Coagulated protein and insoluble contamination were eliminated by centrifuging in a MiniSpin® Plus (Eppendorf) at 14,500 rpm for 15 min at room temperature. Muropeptides contained in the soluble fraction were mixed with 1/3 volume of 0.5 M sodium borate buffer (pH 9.0) and reduced with excess sodium borohydride (NaBH_4_) for 30 min at room temperature. The excess borohydride was neutralised with phosphoric acid (dilution 1:10) to pH 3–4. Finally the samples were filtered through Millex®-GV Filter 0.22 μm (Millipore) units and stored at −20 °C. Reduced muropeptides were separated and analysed by HPLC (Breeze™ 2 System, Waters). Elution products were detected at wavelength 204 nm and identified by the retention time obtained. The relative quantity of muropeptides present in each sample was determined by integration of their respective absorption areas (Breeze™ 2, Waters) and expressed as a molar fraction (mol%) of the total content. When required, the individual peaks were collected, vacuum dried and stored at −20 °C.

### Quantification of muropeptides

Individual muropeptide M5 (monomer disaccharide pentapeptide), M5N (anhydrous monomer disaccharide pentapeptide), D45 (dimer disaccharide tetrapeptide-pentapeptide) and D45N (anhydrous dimer disaccharide tetrapeptide-pentapeptide), natural substrates necessary for enzymatic digestion and kinetics assays, were recovered from the full HPLC pattern of reduced muropeptides, desalted and concentrated from *E. coli* DV900 (a mutant with deletion of nine PBPs and depleted D,D-carboxypeptidase and D,D-endopeptidase activities). The concentration of each product was quantified as a function of the *meso*A_2_pm (DAP) amino acid content using the methodology described by Work [31].

### Lysogenization in *E. coli* DV900. Transformation and expression of recombinant clones

*E. coli* DV900(DE3) was constructed using the λDE3 Lysogenization Kit (Novagen). The transformation of each recombinant clone (pET-PBP4HNC, pET-PBP4HN, pET-PBP4HC) in competent lysogenic cells was carried out by a thermal shock protocol, while overexpression of recombinant proteins was done as described above for clones transformed in *E. coli* BL21(DE3).

### *In vivo* PBP4 D,D-peptidase activities on macromolecular peptidoglycan of *Escherichia coli*

*In vivo* studies of D,D-peptidase activities (D,D-carboxypeptidase; D,D-endopeptidase) for LMM-PBP4 on macromolecular peptidoglycan of *Escherichia coli* was carried out using bacterial cultures of recombinant constructs pET-PBP4HNC, pET-PBP4HN, pET-PBP4HC, transformed into a lysogenized mutant strain *E. coli* DV900(DE3), cultured with and without IPTG induction. These samples were used in the preparation and separation of peptidoglycan, according to the protocols mentioned above. The relative amount of monomer M5 and dimer D45 in the murein *sacculus* of the mutant strain *E. coli* DV900 allowed us to investigate *in vivo* the D,D-carboxypeptidase function (on M5) and the D,D-endopeptidase-D,D-carboxypeptidase function (on D45) for LMM-PBP4. Their activity was estimated from the variation in the abundance of the substrate and product muropeptides relative to a control sample in the HPLC analyses of digested *sacculi*. The results correspond to the mean value of three individual experiments.

### *In vivo* PBP4 D,D-peptidase activities on macromolecular peptidoglycan of *Pseudomonas aeruginosa*

For *in vivo* studies of D,D-peptidase activities (D,D-carboxypeptidase; D,D-endopeptidase) of LMM-PBP4 on macromolecular peptidoglycan of *Pseudomonas aeruginosa* were used murein preparations of the wild-type reference strain PA14WT under natural conditions and after antibiotic treatment. Transformant strain PA14WT/pHERD-PBP4 was used without induction, under overexpression conditions [L(+)arabinose 0.2 %] and after antibiotic inactivation for LMM-PBP4. LMM-PBP4 inactivation was carried by treatment of the culture with cefoxitin (10 μg/ml) at 37 °C for 42 min (one mass doubling). Enzyme activities were estimated from the variation in the abundance of presumed substrate and product muropeptides relative to a control sample in the HPLC analyses. The results correspond to the mean value of three independent experiments.

### D,D-peptidase activity on natural substrates. Kinetic studies

The D,D-carboxypeptidase activity for LMM-PBP4 of PAO1 was assayed in vitro by monitoring the appearance of the monomeric tetrapeptide (M4) in mixtures containing increasing concentrations of monomer disaccharide pentapeptide (M5) (14.6 μM to 227.0 μM) as substrate, purified enzyme PBP4HC (0.57 μM) and PBS 1X pH 8.0, in a final volume of 200 μl. Reaction mixtures were incubated at 37 °C for 150 min. D,D-endopeptidase activity was determined using increasing concentrations of the dimeric compound tetrapentapeptide (D45) (from 8.3 μM to 137.3 μM), PBP4HC protein (0.59 μM) and PBS 1X pH 8.0. Reaction mixtures were incubated at 37 °C for 110 min. All enzyme reactions were terminated by boiling the samples for 2 min; they were centrifuged at 14,500 rpm for 10 min, filtered (Millex-GV Filter 0.22 μm, Millipore) and analysed by HPLC. Apparent *K*_*m*_ and *V*_*max*_ values were obtained from double-reciprocal Lineweaver-Burk plots of the data. *k*_cat_ was determined as *V*_*max*_/[E_0_] (where [E_0_] corresponds to the micromolar concentration of purified LMM-PBP4). Graphical and statistical analyses were performed using the GraphPad Prism® v5.01 programme (GraphPad Software, Inc.). The results correspond to the mean value of experiments done in triplicate.

### Three-dimensional structure

A 3D model of PBP4 from *Pseudomonas aeruginosa* was generated using homology modelling procedures and the crystal structure of PBP4a from *Bacillus subtilis* as template (PDB code: 2J9P) [32]. Model coordinates were built using the SWISS-MODEL server (available at http://swissmodel.expasy.org) and their structural quality was checked using the analysis programmes provided by the same server (Anolea/GROMOS). Global model quality estimation scores are QMEAN4 *raw score*: 0.525 and QMEAN4 *Z-score*: −0.404; these are within the accepted range for homology-based structure models. To optimize geometries, the model was energy-minimized using the GROMOS96_43B1 force field implemented in DeepView v4.1 (Swiss-PdbViewer), using 500 steps of steepest descent minimization followed by 500 steps of conjugate-gradient minimization. To evaluate the enzyme-substrate interaction we used the molecular structure of a fragment of synthetic pentapeptide ligand (AMV-L-Ala-FGA-L-Lys-D-Ala-D-Ala) extracted from the PDB 3ITB archive [33]; this was modified by combined use of the Corina v3.4, Autodock 4.2 [34] and PyMOL v1.6 programmes, followed by manual edition of the atom coordinates to refine the interaction.

## Results

### The cloned molecular forms of LMM-PBP4 from PAO1 are functional as Penicillin-Binding Proteins

**Fig. 1:**
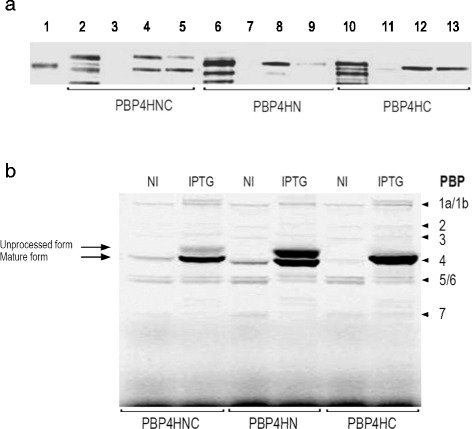
**a** Purification of LMM-PBP4. SDS-PAGE analysis of purified recombinant proteins PBP4HNC (lanes 2, 3, 4, 5), PBP4HN (lanes 6, 7, 8, 9) and PBP4HC (lanes 10, 11, 12, 13). Lane 1, molecular weight marker (51 kDa); lanes 2, 6 and 10, flow-through; lanes 3, 7 and 11, elution with 125 mM imidazole from Ni-NTA; lanes 4, 8 and 12, elution with 250 mM imidazole from Ni-NTA; lanes 5, 9 and 13, elution with 500 mM imidazole from Ni-NTA.. **b** Identification assays for LMM-PBP4. Pattern of Bocillin™ FL binding to membrane protein extracts prepared from strains *E. coli* BL21(DE3)/pET-PBP4HNC, *E. coli* BL21(DE3)/pET-PBP4HN and *E. coli* BL21(DE3)/pET-PBP4HC. Extracts from non induced cell (NI) and induced by IPTG (IPTG) are shown. Unprocessed and mature forms of the overexpressed proteins are indicated by arrows. PBPs profile model (PBP) for *Escherichia coli* is shown

The constructs for LMM-PBP4 of *Pseudomonas aeruginosa* PAO1 [pET-PBP4HNC (PBP4HNC, NC-terminal His · tag), pET-PBP4HN (PBP4HN, N-terminal His · tag) and pET-PBP4HC (PBP4HC, C-terminal His · tag)], enabled us to evaluate the yield and behaviour of the whole recombinant product, maintaining the sequence which defines its signal peptide, avoiding alterations to the structural nature of this protein and allowing it to be recovered in functional and constitutive conditions very close to those of its native organisation. After induction, overexpression and subsequent purification assays, SDS-PAGE and western blot analyses confirmed that only one protein migrated to a position close to 51 kDa, matching the molecular masses expected and calculated for the constructs pET-PBP4HNC (55.54 kDa), pET-PBP4HN (54.02 kDa) and pET-PBP4HC (53.38 kDa) (Fig. 1a). Although the final products for the recombinant proteins developed are identical, the forms adopted during synthesis vary and an unprocessed variant can be recognised with a higher molecular mass than the mature form (processed protein) in recombinant proteins PBP4HNC and PBP4HN. In both cases the induced material presents two bands (upper band, unprocessed protein; lower band, processed protein), while for PBP4HC only one band is identified, which corresponds to the mature form of the recombinant protein (Fig. 1b). The recombinant product recovered from the purification assays is the mature form of the protein for the clone pET-PBP4HC, and the unprocessed and processed forms for the clones pET-PBP4HNC and pET-PBP4HN. This allows us to evaluate the presence of the His · tag bound to the amino terminal and the difficulty which this configuration causes in protein maturation. Both the precursor and the mature forms present the ability to bind to BOCILLIN™ FL.

### *In vivo* D,D-peptidase activity of LMM-PBP4 on macromolecular peptidoglycan of *Escherichia coli*

**Fig. 2:**
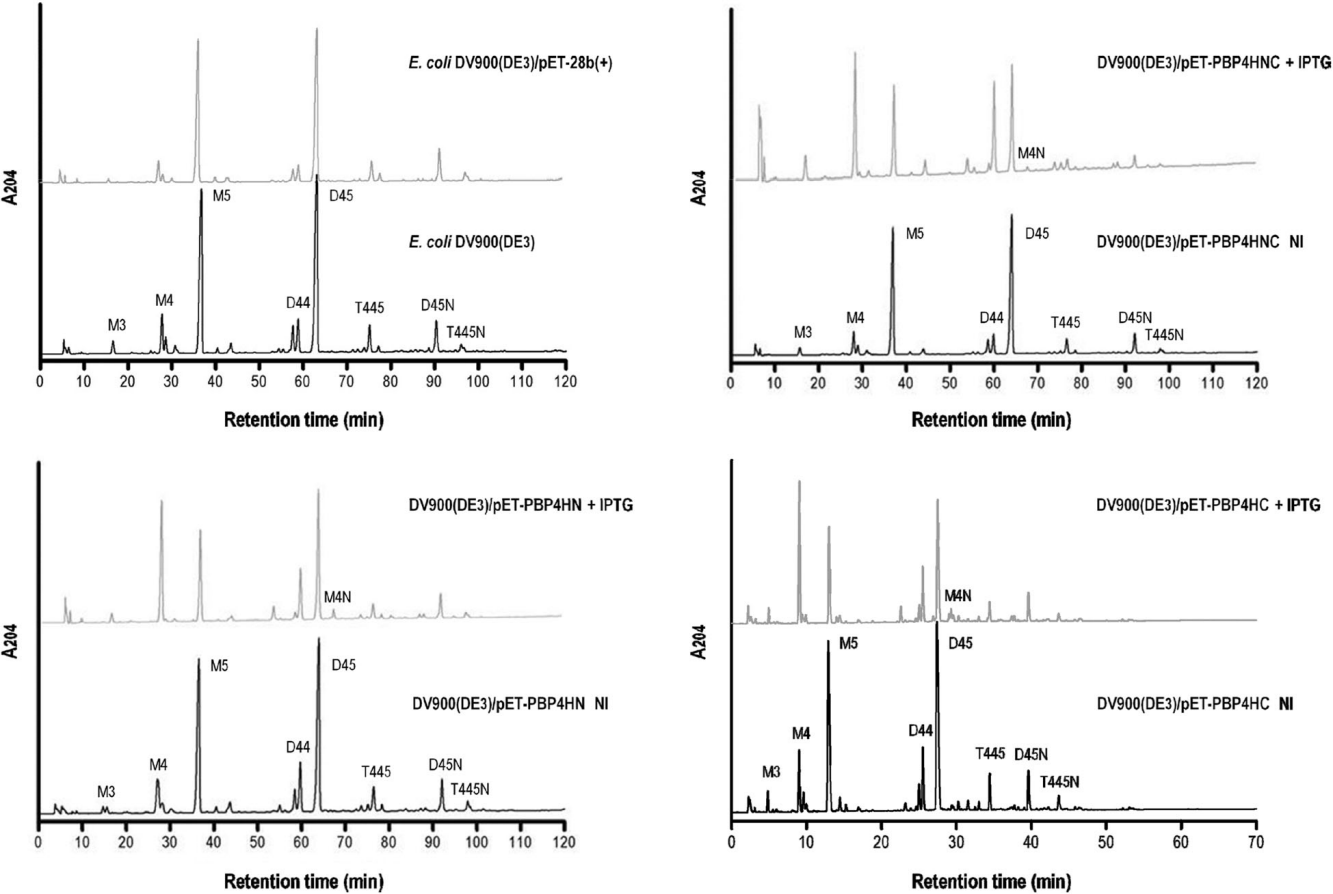
D,D-peptidase activities on peptidoglycan of *Escherichia coli*. HPLC chromatograms of peptidoglycan obtained from strains *E. coli* DV900(DE3)/pET-PBP4HNC, *E. coli* DV900(DE3)/pET-PBP4HN and *E. coli* DV900(DE3)/pET-PBP4HC, uninduced (NI) and induced by IPTG (IPTG). Chromatograms for the lysogenized mutant strain *E. coli* DV900(DE3) and transformed with the expression vector pET-28b(+) are presented as controls. The following muropeptides are identified: M3, monomer disaccharide tripeptide; M4, monomer disaccharide tetrapeptide; M5, monomer disaccharide pentapeptide; M4N, anhydrous monomer disaccharide tetrapeptide; D44, dimer disaccharide tetrapeptide-tetrapeptide; D45, dimer disaccharide tetrapeptide-pentapeptide; D45N, anhydrous dimer disaccharide tetrapeptide-pentapeptide; T445, trimer tetrapeptide-tetrapeptide-pentapeptide; T445N, anhydrous trimer tetrapeptide-tetrapeptide-pentapeptide. A204, absorbance at 204 nm, arbitrary units

D,D-peptidase activities were evaluated *in vivo* on macromolecular peptidoglycan derived from a mutant of *E. coli* (DV900) lacking D,D-carboxypeptidase and D,D-endopeptidase activities and modified by lysogenization, as shown in Fig. 2. The chromatograms obtained for transformant lysogens not induced and induced by IPTG confirm significant decrease of the substrates M5 and D45, in each transformant strain subjected to induction, in comparison with the absence of changes in the muropeptides profile for non-induced material. The muropeptides which determine a D,D-endopeptidase function are the monomers M5 and M4, generated from substrate D45. In this case there is a clear reduction of the substrate and an increase in the amount of M4 in particular. This effect is determined by the use of M5 as an alternative substrate for the second activity of the enzyme, D,D-carboxypeptidase. D,D-carboxypeptidase activity expressed with substrates D45 and M5 favours production of D44 and M4 respectively; this fact amplifies the final amount of M4 monomeric product both directly and indirectly through the occurrence of new D,D-endopeptidase activity on the newly generated dimeric product-substrate D44.

### *In vivo* D,D-peptidase activity of LMM-PBP4 on macromolecular peptidoglycan of *Pseudomonas aeruginosa*

**Fig. 3:**
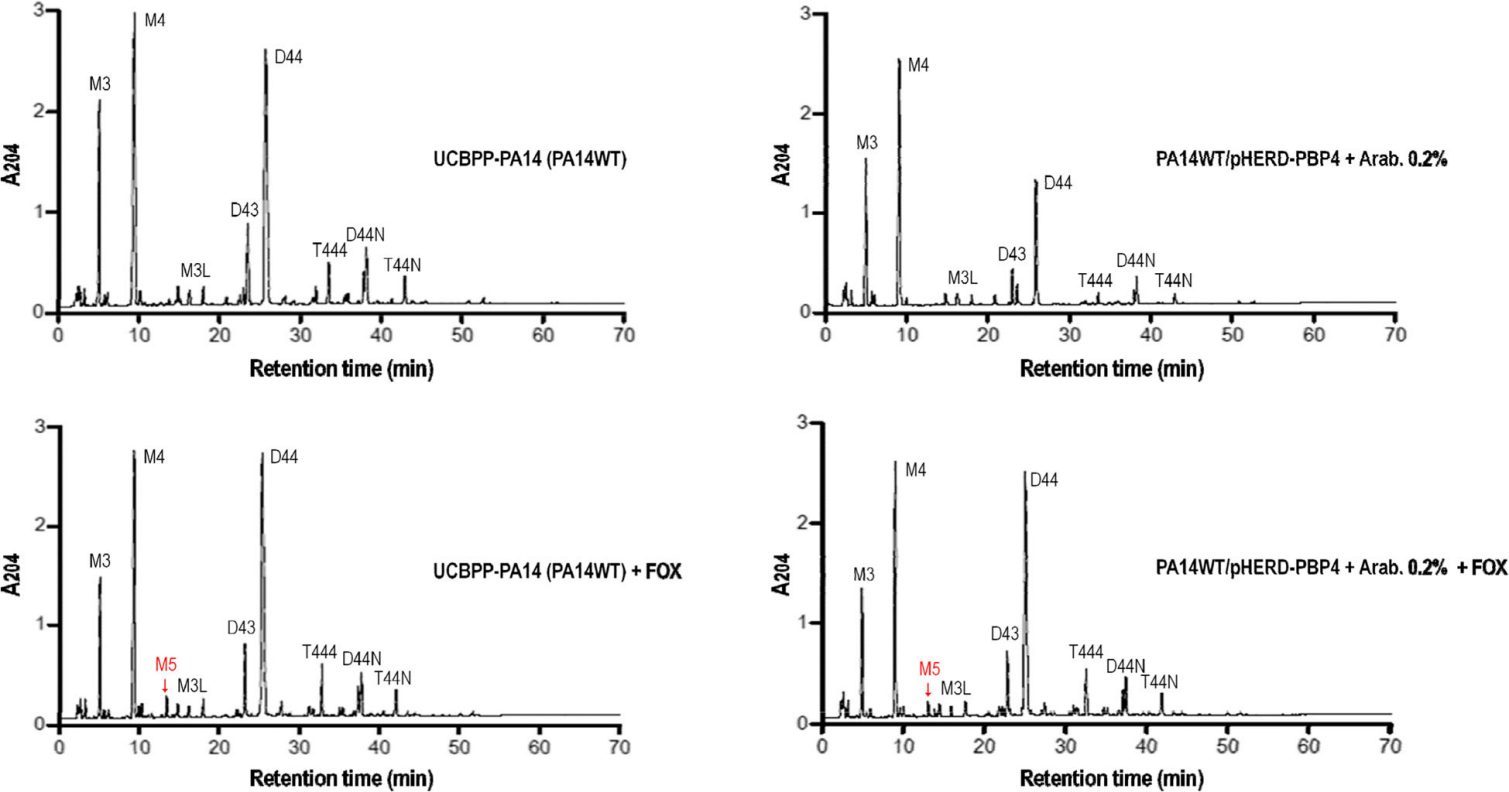
D,D-peptidase activities on peptidoglycan of *Pseudomonas aeruginosa.* HPLC chromatograms of peptidoglycan obtained from reference strain UCBPP-PA14 (PA14WT), PA14WT/pHERD-PBP4 induced with L(+)-arabinose 0.2 %, PA14WT treated with cefoxitin (FOX), and PA14WT/pHERD-PBP4 induced with L(+)-arabinose 0.2 % and treated with cefoxitin (FOX). The following muropeptides are identified: M3, monomer disaccharide tripeptide; M4, monomer disaccharide tetrapeptide; M5, monomer disaccharide pentapeptide; M3L, monomer disaccharide tripeptide associated with lipoprotein; D43, dimer disaccharide tetrapeptide-tripeptide; D44, dimer disaccharide tetrapeptide-tetrapeptide; D44N, anhydrous dimer disaccharide tetrapeptide-tetrapeptide; T444, trimer tetrapeptide-tetrapeptide-tetrapeptide; T444N, anhydrous trimer tetrapeptide-tetrapeptide-tetrapeptide. Arab., arabinose; A204, absorbance at 204 nm, arbitrary units

The peptidoglycan structure for the bacterial model *Pseudomonas aeruginosa* UCBPP-PA14 was evaluated under three conditions, native, LMM-PBP4 overexpression and antibiotic inactivation, as a function of the relative amount of global muropeptides (molar fraction for monomeric, dimeric and trimeric constituents), unitary components (lipoprotein, anhydrous form, monomer disaccharide pentapeptide, DAP-DAP bonds) and related structural parameters (chain length, D-D/total and crosslinking) described in Additional file 2: Table S1 (differences greater than 10 % in the structural values obtained for strains subjected to experimentation as compared to the amounts found in reference strains were considered significant). Under conditions of non-expression of the *dacB* gene and absence of β-lactam (cefoxitin) antibiotic treatment, the peptidoglycan composition for the transformant strain PA14WT/pHERD-PBP4 as compared to the murein composition of the reference strain UCBPP-PA14 (PA14WT) presents a fall in the levels of dimers, trimers, DAP-DAP and D-D/total; these define an increase in the amount of monomers and a lower percentage of crosslinking. There was also a reduction in anhydrous forms which explains a moderate increase in the chain lengths. These effects may be interpreted as a consequence of a basal level of expression of the *dacB* gene in this construction. The peptidoglycan of the transformant strain PA14WT/pHERD-PBP4 under induction [L(+)-arabinose 0.2 %] presents an organisation which reveals a significant decrease in the amount of dimeric and trimeric constituents (PA14WT/pHERD-PBP4, dimers: 21.9 mol% against 29.4 mol%; trimers: 1.2 mol% against 1.9 mol%), leading to a proportional increase in the relative abundance of monomers (PA14WT/pHERD-PBP4, monomers: 76.8 mol% against 67.5 mol%) and a considerable decrease in the crosslinking levels (PA14WT/pHERD-PBP4, crosslinking: 24.3 % against 33.4 %); the reduction in the number of anhydrous forms is also considerable (PA14WT/pHERD-PBP4, anhydrous forms: 4.4 against 7.3), which in turn defines greater chain length (PA14WT/pHERD-PBP4, chain length: 22.2 against 13.6), in the muropeptides composition for the murein of the corresponding non-induced control strain. DAP-DAP and lipoprotein levels do not present major changes, the percentages of D-D/total are slightly higher, while the values assigned to monomer disaccharide pentapeptide present only a slight reduction (PA14WT/pHERD-PBP4, pentapeptide: 0.3 against 0.4). Under antibiotic treatment with cefoxitin (FOX), the structural composition of PA14WT peptidoglycan presented an important increase in the relative quantity of monomer disaccharide pentapeptide (M5) (PA14WT, pentapeptide: 3.7 mol% against 0.4 mol%) which stimulates the formation of dimers and trimers with 4–3 bonds (if the DAP-DAP levels are not affected) (PA14WT, dimers: 37.3 mol% against 32.6 mol%; trimers: 3.4 mol% against 2.4 mol%), and as a consequence of this increase there is a reduction in monomers (PA14WT, monomers: 59.1 mol% against 63.9 mol%) and an increase in the crosslinking percentage (PA14WT, crosslinking: 44.5 % against 37.6 %). The consequence of this efficient blocking of all the LMM-PBPs by the β-lactam cefoxitin, interrupting D,D-carboxypeptidase and D,D-endopeptidase functions, is favourable to the results described. In the case of LMM-PBP4 induction [L(+)-arabinose 0.2 %] and treatment with cefoxitin, the peptidoglycan architecture of the PA14WT/pHERD-PBP4 strain presents structural parameters very close to those reported in the wild-type (cefoxitin inhibits the function of LMM-PBP5/6 and LMM-PBP7 and is incapable of blocking the amount of protein expressed); this confirms that there is a reversion, as there is again a rise in the level of monomers and a reduction in crosslinking, defined by the expression of D,D-endopeptidase activity. The recovery in the pentapeptide (M5) value is not very efficient, since LMM-PBP4 of *Pseudomonas aeruginosa* appears to be better for D,D-endopeptidase than D,D-carboxypeptidase (Fig. 3).

### D,D-peptidase activity of LMM-PBP4 on natural muropeptides. Kinetic evaluation

**Fig. 4:**
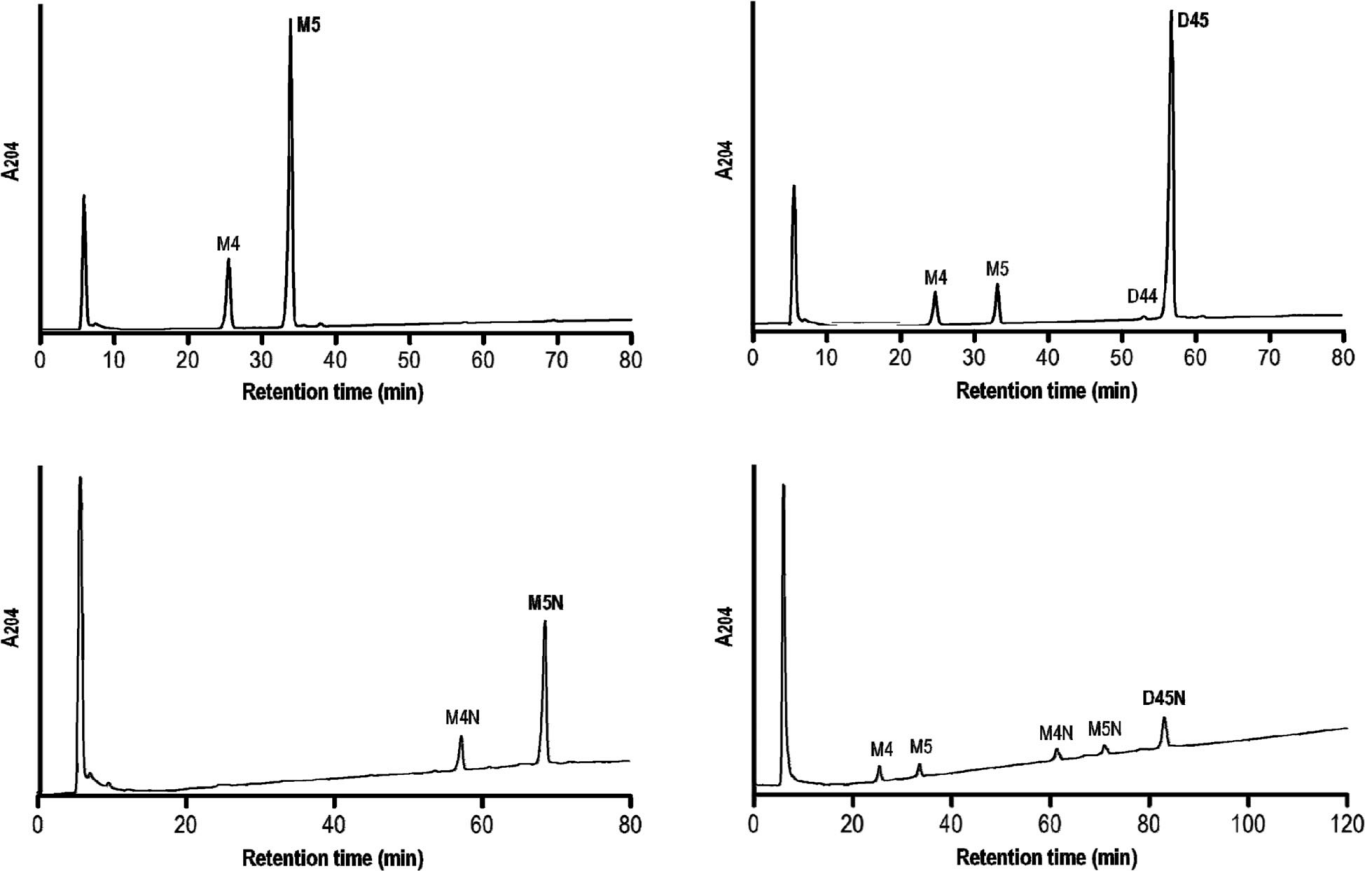
D,D-peptidase activity on purified muropeptides. HPLC chromatograms for digestion assays with purified recombinant protein PBP4HC on natural substrate, M5 (monomer disaccharide pentapeptide), D45 (dimer disaccharide tetrapeptide-pentapeptide), M5N (anhydrous monomer disaccharide pentapeptide) and D45N (anhydrous dimer disaccharide tetrapeptide-pentapeptide). The identified products are labeled: M4, monomer disaccharide tetrapeptide; D44, dimer disaccharide tetrapeptide-tetrapeptide; M4N, anhydrous monomer disaccharide tetrapeptide. A204, absorbance at 204 nm, arbitrary units

The enzyme digestion assay for PBP4HC on the purified and concentrated muropeptides M5 (monomer disaccharide pentapeptide) and D45 (dimer disaccharide tetrapeptide-pentapeptide), and the evaluation carried out with HPLC, confirm the capability of this enzyme to develop D,D-carboxypeptidase activity, since it can remove a D-alanine terminal from the peptide chain for the substrate M5 and generate an increase in the product M4 (monomer disaccharide tetrapeptide). The protein studied for its effects on dimer disaccharide D45 demonstrates its competence as an efficient D,D-endopeptidase, hydrolysing the peptide bond which maintains the dimer configuration of this substrate and recovering the expected monomer products, M5 and M4 respectively. The amount of monomer disaccharide tetrapeptide was slightly higher than the amount of monomer disaccharide pentapeptide recovered, which demonstrates secondary D,D-carboxypeptidase activity from the M5 generated; the same function is demonstrated by the production of D44 (dimer disaccharide tetrapeptide-tetrapeptide) from the original substrate D45. Enzyme activity on anhydrous derivates M5N (anhydrous monomer disaccharide pentapeptide) and D45N (anhydrous dimer disaccharide tetrapeptide-pentapeptide) shows very similar behaviour to that of muropeptide substrates with no chain terminus residues, although with a minority product profile. This helps to explain the D,D-carboxypeptidase and D,D-endopeptidase properties of LMM-PBP4 (PAO1), Despite this, D,D-carboxypeptidase activity can be identified on substrate M5 from the presence of product M4N, as well as D,D-endopeptidase/D,D-carboxypeptidase bifunctionality on the dimer substrate D45N, from the recovery of muropéptidos M5, M4N and M5N (products indicating D,D-endopeptidase activity) and monomer M4 (product derived from direct D,D-endopeptidase activity on natural substrate D45N and D,D-carboxypeptidase activity on the monomer product M5) (Fig. 4). Analysis of D,D-carboxypeptidase/D,D-endopeptidase activity for LMM-PBP4 on purified muropeptides M5 and D45 yielded kinetic constants *V*_*max*_, *K*_*m*_ and *k*_*cat*_. A lower value was found for *K*_*m*_ on the dimer substrate D45, as compared to the monomer substrate M5 (> *K*_*m*_); furthermore the *k*_*cat*_ levels were 15–17 times higher for activity on substrate D45, as compared to the activity shown on natural substrate M5 (Additional file 2: Table S2).

### 3D structural homology analysis of LMM-PBP4 revalidates D,D-endopeptidase activity

**Fig. 5:**
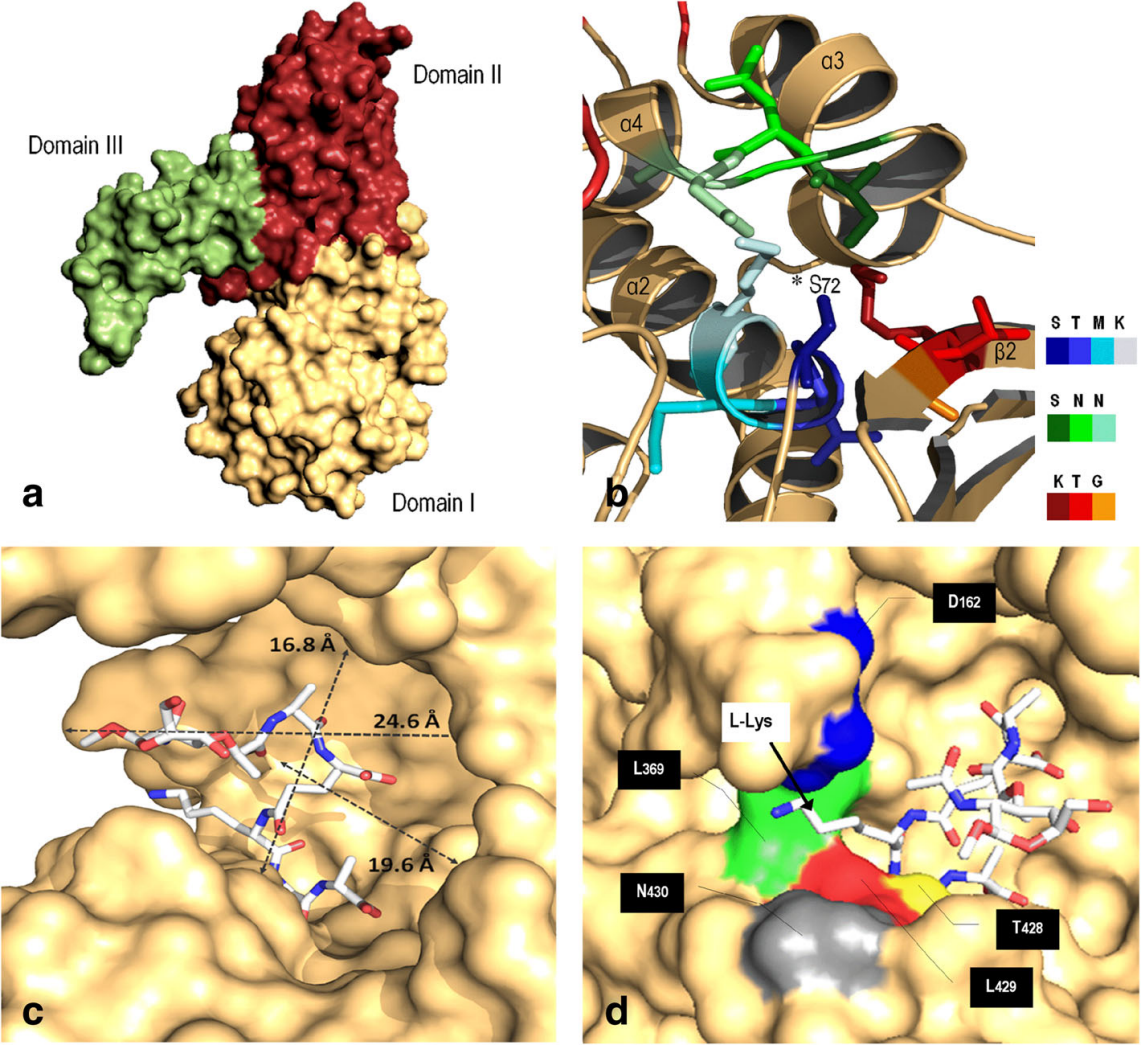
3D model for LMM-PBP4 of PAO1. **a** Homology modeling of monomer *Pseudomonas aeruginosa* LMM-PBP4 (surface representation). The different domains are presented in brown (domain I), red (domain II) and green (domain III). **b** Domain I and conserved motifs in the active site of LMM-PBP4. For the first conserved sequence SxxK (STMK; *S72, catalytic serine at position 72), located at the beginning of α2 helix, residues are represented by an intense, blue, cyan and pale blue color, respectively. The second conserved sequence SxN (SNN), located in a short loop between α3-α4, is represented by a gradient of colors derived from green. The three residues of the third conserved motif KTG, located in a β sheet (β2) are represented on dark red, red and orange, respectively. **c** Location for synthetic peptide AMV-L-Ala-FGA-L-Lys-D-Ala-D-Ala (linear representation) is indicated within the active site model for LMM-PBP4. Estimated distances for depth, height and width in this cavity are shown. **d** Putative residues constituents for the specific subsite in the active site of LMM-PBP4. Location of the amino acid lysine (L-Lys) of the synthetic substrate AMV-L-Ala-FGA-L-Lys-D-Ala-D-Ala, the residue equivalent to *meso*A_2_pm in natural muropeptides, is indicated. Each amino acid has been labeled and highlighted by a color (aspartic acid 162, *blue*; leucine 369, *green*; threonine 428, *yellow*; leucine 429, *red*; asparagine 430, *gray*). AMV, methyl 2-(acetylamino)-3-O-[(1R)-1-carboxyethyl]-2-deoxy-beta-D-glucopyranoside; FGA, gamma-D-glutamic acid

Homology simulation for the protein LMM-PBP4 of *Pseudomonas aeruginosa* O1 (Fig. 5a) enables us to distinguish clearly the three motives which define it as LMM-PBP class C, subclass C1, located in domain 1. As in the case of its structural homologues, PBP4a of *Bacillus subtilis* and D,D-Peptidase of *Actinomadura* R39, and its functional homologue PBP4 of *Escherichia coli*, for the monomer unit of LMM-PBP4 in PAO1 we confirmed the presence of an amino acid insert which constitutes domains II and III (with domain III contained in domain II). The active site of the protein is located in domain I (PB domain), at the interface between 5 β sheets and a group of α helices. It consists of the initial segment of helix α2 (S72-K75) which is part of the STMK sequence [which in turn includes the catalytic serine in position 72 (* S72)], the lateral chain of a β2 sheet (V424-L429) which contains the KTG conserved motive, and a small loop which connects helices α3-α4 (Y314-N317) and incorporates a third SNN conserved sequence (Fig. 5b). Residues from domains II and III take part of the roof of a cavity which communicates at depth with the inner catalytic serine. The estimated dimensions of this U-shaped slit on the surface of the protein (depth 24.6 Å, width 19.6 Å) are very close to those described for the slit of the active site on the functional homologue PBP4 of *E. coli* (depth 20 Å, width 15 Å) (Fig. 5c) [35]. Molecular simulation of the coupling of the mimetic pentapeptide substrate AMV-L-Ala-FGA-L-Lys-D-Ala-D-Ala in the cavity of the active site for LMM-PBP4 of PAO1 validates its size and tolerance of larger ligands; it also allows us to identify the constituent amino acids of a sub-site in the catalytic pocket [aspartic acid 162 (D162), leucine 369 (L369), threonine 428 (T428), leucine 429 (L429) and asparagine 430 (N430)] (Fig. 5d). They are related (as described for equivalent amino acids in homologous proteins) with the specificity for its substrate, by recognition of the terminal group NH_3_^+^-CH-COO^−^ for the amino acid *meso*A_2_pm of the peptide chain. Actually, in the molecular model, the L-Lys of the mimetic pentapeptide substrate is linked to the protein by specific interaction with three of those residues: the carbon chain of L-Lys interact with leucine 369 (L369), and the NH_3_^+^ terminal of L-Lys interact with the COO^−^ group of aspartic acid 162 (D162) (mainly) and the CO group of asparagine 161 (N161).

## Discussion

In *Pseudomonas aeruginosa*, the inactivation of AmpD and point mutations in AmpR and DacB (LMM-PBP4) have been found to lead to AmpC overexpression; the shared aspect of each of these three proteins is the functional requirement for concomitant maturation of its peptidoglycan. These two processes (AmpC overexpression and peptidoglycan maturation) are directly interconnected by the pathway for the efficient recycling of muropeptides [36–38]. AmpD and DacB are not required for overexpression. A functional AmpR is required, but on the contrary, it is the mutational inactivation of AmpD or DacB what produces the overexpression. Recent studies stressed the complexity of this relationship, in which the role of the amidase AmpD is definitely known, the role of the transcriptional regulator AmpR is partially recognised with some degree of certainty, while researchers have speculated on the function of LMM-PBP4 based on information available for coding gene of this protein in the PAO1 and UCBPP-PA14 strains. The putative nature of this information has been partly resolved in recent research showing, for example, that LMM-PBP4 of *Pseudomonas aeruginosa* exercises control over the function of AmpR, presumably as a result of its ability to create or destroy a particular muropeptide chain subunit [21, 23, 38]. A study aimed to characterize the role of LMM-PBPs in peptidoglycan composition, β-lactam resistance, and AmpC regulation, indicated that PBP4 play a significant role as D,D-carboxipeptidase only when PBP5 is absent; on the other hand, the peptidoglycan structure of PBP4 and PBP7 single and double mutants showed that these proteins have D,D-endopeptidase activity [39]. A recent publication explored the catalytic capability of this enzyme through direct digestion assays on peptidoglycan *sacculus* and a variety of synthetic substrates, finding that LMM-PBP4 (soluble form with no signal peptide) exerts D,D-carboxypeptidase and D,D-endopeptidase activity, just like its functional homologue PBP4 of *Escherichia coli* (although this protein is not involved in resistance to antibiotics) [38]. In an attempt to confirm the above with a dynamic experimental model *in vivo* using natural substrates, we investigated the function for LMM-PBP4 of *Pseudomonas aeruginosa* initially using digestion assay on macromolecular peptidoglycan derived from *E. coli* DV900(DE3), a lysogenized mutant strain (CS802-2 Δ*pbp4B*) with deletions in coding gene for all class C LMM-PBPs, as well as AmpC. This provided a biological system with structural organisation of the cell wall which was particularly favourable for studying D,D-carboxypeptidase and D,D-endopeptidase activities. Variations in the relative abundance of muropeptides enabled us to identify a significant diminution of the substrates M5 and D45, in each transformant strain subjected to induction, in comparison with the absence of changes in the muropeptides profile for non-induced material. Starting with substrate D45, the increase in the monomeric products M5 and particularly M4 (effect determined by the provision of M5 as an alternative substrate for secondary D,D-carboxypeptidase activity) define a D,D-endopeptidase function for LMM-PBP4. This does not appear to be affected by the presence of a D-alanine residue in position 5 for an acceptor peptide chain in dimeric muropeptides. The presence of the minority product D44 (new D,D-endopeptidase activity on the dimeric substrate D44 amplifies the final amount of monomeric product M4) and M4 confirms D,D-carboxypeptidase activities on substrates D45 and M5 respectively. The lower relative abundance of D44, compared to the levels of M5 and M4 recovered from substrate D45, eliminates the possibility that LMM-PBP4 of PAO1 carries out an initial D,D-carboxypeptidase activity. A smaller reduction in D45N (anhydrous dimer disaccharide tetrapeptide-pentapeptide), T445 (trimer tetrapeptide-tetrapeptide-pentapeptide) and T445N (anhydrous trimer tetrapeptide-tetrapeptide-pentapeptide) represents an important extension of substrate specificity in both activities (particularly D,D-endopeptidase activity). It has been suggested that the presence of 1,6-anhydroMurNAc residue might have a significant implication for D,D-carboxypeptidase/D,D-endopeptidase eactions, given the smaller reduction in anhydrous D45N-T445N substrates under muropeptides D45-T445. This hypothesis would appear to be incorrect, in view of the presence of a smaller but detectable amount of product M4N (proceeding from D,D-endopeptidase activities for anhydrous dimeric substrate D45N and D,D-carboxypeptidase activities on anhydrous monomer M5N) in each muropeptides profile generated from induced material. The above shows the simultaneous bifunctionality of D,D-endopeptidase/D,D-carboxypeptidase exerted *in vivo* on macromolecular peptidoglycan for each of the recombinant proteins for LMM-PBP4 of PAO1 (PBP4HNC, PBP4HN and PBP4HC) expressed in the strain of *Escherichia coli* tested. Cloning of the coding gene for LMM-PBP4 of PAO1 in expression vectors compatible with *Pseudomonas aeruginosa* enabled us to design an experiment to investigate the modulating role of this LMM-PBP in the molecular integrity of the cell wall for this bacterial model. The organisation of the peptidoglycan for the wild-type strain UCBPP-PA14, containing the recombinant construct for LMM-PBP4 (pHERD-PBP4), under induction shows a reduction in the amount of dimeric and trimeric constituents, leading to a proportional increase in the quantity of monomers and an important reduction in crosslinking, as well as a fall in the number of anhydrous forms and a smaller reduction in the levels of M5 (monomer disaccharide pentapeptide). These results are consistent with the occurrence of a main D,D-endopeptidase activity and a secondary D,D-carboxypeptidase activity, produced by the overexpression of LMM-PBP4 in this bacterial system; the same was demonstrated in a previous study, in which the effect of LMM-PBP4 overproduction on the murein synthesized *in vivo* for the model *Escherichia coli* presented results with a similar tendency, with low level of crosslinking in (DD)-D-Ala-DAP peptide bridges, an increase in the quantity of monomers and a reduction in the number of dimers, trimers and tetramers. This composition reflects the exclusive D,D-endopeptidase/D,D-carboxypeptidase functions exercised by LMM-PBP4 of *E. coli* and involved in the turnover of the peptide crosslinking of their peptidoglycan [40]. A third group of studies, intended to evaluate direct D,D-peptidase activity for the purified PBP4HC on natural muropeptides M5, D45, M5N and D45N, confirm that LMM-PBP4 (PAO1) is capable of exert D,D-endopeptidase/D,D-carboxypeptidase catalytic functions. Jointly with these digestion assays, and as a way of establishing a definite conclusion on the predominant enzyme activity for LMM-PBP4, we investigated the individual behaviour of each activity by estimating the kinetic parameters *V*_*max*_, *K*_*m*_ and *k*_*cat*_. A constant *K*_*m*_ of 71.9 ± 2.1 μM for the D,D-carboxypeptidase activity of LMM-PBP4 on natural substrate M5, as compared to an estimated value for *K*_*m*_ (*K*_*m*_ 20.4 ± 1.6 μM) for PBP4 of *Escherichia coli* (functional orthologue for LMM-PBP4 of PAO1) on synthetic substrate N-acetylmuramyl-pentapeptide (structurally closer to M5) [13], defines lower D,D-carboxypeptidase behaviour for LMM-PBP4. The identification of lower values of *K*_*m*_ of LMM-PBP4 (PAO1) for D45 reflects the greater affinity between this protein and its dimeric substrate, and a minor presence of the elution product D44, exclude the initial occurrence of D,D-carboxypeptidase activity. These data, together with the much higher catalytic effectiveness values (*k*_*cat*_) found for LMM-PBP4 activity on dimeric substrate D45 than the *k*_*cat*_ values for this protein on natural substrate M5, confirm that D,D-endopeptidase activity is predominant for LMM-PBP4 of *Pseudomonas aeruginosa* O1. Finally, analysis of the structural model for the monomeric unit of LMM-PBP4 in PAO1 evidence a trimodular organisation of this enzyme (presence of a transpeptidase domain associated with two other domains), with a Russian doll (*matryoshka*) integration model for each module (the third domain included in the second which is itself contained in the first) and a very similar orientation to that described for structural homologues PBP4a of *Bacillus subtilis*, D,D-peptidase of *Actinomadura* R39 and the functional homologue PBP4 of *Escherichia coli* [32, 35, 41]. On the back face of domain II, opposite the contact surface with domain I (PB), three residues of lysine and four of arginine expose a positively charged area and form a bonding mechanism to the internal membrane which is an alternative to the electrostatic and polarity interactions defined for the carboxyl terminal in this protein. A similar surface has been described for domain II of PBP4a of *B. subtilis* and D,D-peptidase of *Actinomadura* R39, however the presence of residues of this kind has not been recognised for LMM-PBP4 in the *Escherichia coli* bacterial system [32, 41]. Domain III with a less rigid architecture, presents a segment which forms an integral part of the active site on this protein. Comparison with related enzymes shows that domain I for LMM-PBP4 of PAO1 contains the necessary groups to activate catalytic serine S72 (STMK, SNN, KTG) and to carry out a nucleophile attack on the substrate. The residues involved in the acetylation and deacetylation mechanisms by PBPs have not been clearly identified, however in our case, for the first process, a lysine in position 75 (K75) is probably the priority structural amino acid. The constituent residues of domains II and III determine a cavity which communicates with the catalytic serine of the active centre, with estimated width, depth and height which would favour access of small molecules (antibiotics) and longer substrates, as described in other members of this sub-class of proteins. Our three-dimensional model highlights the orientation and ordering of the residues of domain III at the entrance of the slit to the active site, which appears to support the idea of the function of this domain in steric control of access by the substrate, a competence which has not been confirmed but has been deduced for the homologue protein PBP4 of *E. coli* [35]. The positional equivalence demonstrated between residues in the active site of LMM-PBP4 in PAO1, particularly aspartic acid in position 162 (D162) and asparagine in position 430 (N430), with amino acids conserved and defined as constituents of a specific sub-site in the catalytic pocket of homologue proteins PBP4 of *E. coli* (D155, Q422), PBP4a of *B. subtilis* (D145, S416) and D,D-peptidase in *Actinomadura* R39 (D142, S415), involved in the specificity by their substrate, through recognition of a residue in position 3 (*meso*A_2_pm) of the peptide chain, may constitute a distinguishing precedent, in terms of substrate requirement, between bifunctional proteins with D,D-carboxypeptidase/D,D-endopeptidase activities and monofunctional proteins with D,D-carboxypeptidase or D,D-transpeptidase activities [13]. It should be noted that for the structural model of PBP4a in *B. subtilis*, a threonine in position 394 (T394) constitutes an amino acid residue with the capability of establishing a hydrogen bridge with T412 (conserved motive KTG), contributing to the network of bonds which strengthen the active site. This bonding structure has been recognised in an equivalent position in other representatives of LMM-PBPs, class C, subclass C1. However, in PBP5, PBP6 and PBP6b of *E. coli* (proteins with an exclusive D,D-carboxypeptidase function), an arginine residue occupies this position, acting as a steric impediment to the positioning of a larger substrate than the methyl group of the last D-alanine in the peptide chain (the presence of a small residue like threonine leaves a free space which could receive the peptide chain of a second strand of peptidoglycan) [32]. In our simulation for LMM-PBP4 of *Pseudomonas aeruginosa*, a threonine amino acid (T386) in a position which coincides with the location of T394 in *Bacillus subtilis*, together with the structural data given above, could represent constitutive information highlighting the occurrence of the D,D-endopeptidase function.

## Conclusions

This study confirms *in vivo* the D,D-carboxypeptidase/D,D-endopeptidase bifunctionality of LMM-PBP4 of *Pseudomonas aeruginosa* on macromolecular peptidoglycan in two models, *Escherichia coli* and *Pseudomonas aeruginosa*. It highlights the occurrence of D,D-endopeptidase activity as the principal activity on these substrates. Kinetic assays on natural muropeptides allowed us to confirm both these hydrolytic capabilities and to define catalytic D,D-endopeptidase activity as predominant. This competence is supported structurally by the composition and dimensions of the active site, obtained by molecular modelling of this protein. This description of the function and conformation of LMM-PBP4 constitutes a new perspective which supports the proposal of this protein as a potential hydrolase-autolysin associated with peptidoglycan maturation and recycling, The fact that mutant PBP4 induces AmpC, may indicate that a putative muropeptide subunit product of the DD-EPase activity of PBP4 could be a negative regulator of the pathway, and this makes a significant contribution to further understand the regulatory pathways for the induction and constitutive hyperproduction of chromosomal AmpC β-lactamase on *Pseudomonas aeruginosa*.

## Additional files

Additional file 1:
**Table S1.** Strains used in this study. **Table S2.** Plasmids and phages used in this study. **Table S3.** Oligonucleotides used in this study. (DOCX 25 kb)
Additional file 2:
**Table S1.** HPLC analysis of muropeptides prepared from the peptidoglycan of *Pseudomonas aeruginosa* UCBPP-PA14 grown under natural conditions, with overexpression of LMM-PBP4 and antibiotic inactivation. **Table S2.** Kinetic parameters (*V*_*max*_, *K*_*m*_, *k*_*cat*_) for LMM-PBP4 of *Pseudomonas aeruginosa* O1 on natural substrates M5 and D45. (DOCX 29 kb)
