# Supplementary material for: In vivo functional and molecular characterization of the Penicillin-Binding Protein 4 (DacB) of Pseudomonas aeruginosa
Source: BMC Microbiol. 2016 Oct 6;16:234. doi: 10.1186/s12866-016-0853-x (PMC5054556; doi:10.1186/s12866-016-0853-x)
Supplement: Additional file 1: — Table S1. Strains used in this study. Table S2. Plasmids and phages used in this study. Table S3. Oligonucleotides used in this study. (DOCX 25 kb) [file 12866_2016_853_MOESM1_ESM.docx]

**Table S1.** Strains used in this study.

| Strains | Genotype/relevant characteristics | Source or reference |
| --- | --- | --- |
| *Pseudomonas aeruginosa* |  |  |
| PAO1 | Reference strain | [24] |
| UCBPP-PA14 | Reference strain | [25] |
| *Escherichia coli* |  |  |
| *E. coli* DH5α | F- Φ80d*lacZ*Δ*M15* Δ(*lacZYA-argF*)*U169 deoR supE44* λ- *thi-1* *endA1* *gyrA96* *hsdR17* *recA1* *relA1* | Laboratory collection |
| *E. coli* BL21(DE3) | F- *ompT* *hsdS*_B_ (r_B_-m_B_-) *dcm* *gal* λDE3 | Laboratory collection |
| *E. coli* CS802-2 | CS109 Δ*ponB*, *dacA*, *dacB*, *dacC*, *dacD*, *pbpG*, *ampH*, *ampC* | [26] |
| *E. coli* DV900 | CS802-2 Δ*pbp4B* | [26] |
| *E. coli* HMS174(DE3) | Positive control lysogen λDE3 Lysogenization Kit | Novagen |
| *E. coli* DV900(DE3) | CS802-2 Δ*pbp4B* coinfected with λDE3 prophage | This work |
|  |  |  |

**Table S2.** Plasmids and phages used in this study.

| Plasmids/Phages | Relevant characteristics | Source or reference |
| --- | --- | --- |
|  |  |  |
| pGEM®-T Easy | PCR cloning vector, Ap^r^ | Promega |
| pET-28b(+) | *E. coli* expression vector, Km^r^ | Novagen |
| pHERD26T | *P*_BAD_ promoter, pRO1614 *ori*, pBR322 *ori*, *oriT*, Tc^r^ | [27] |
| λDE3 phage | Recombinant phage carrying the cloned gene for T7 RNA polymerase under *lac*UV5 control | Novagen |
| Helper phage | Phage that allows the integration of λDE3 into (or be excised from) the chromosome | Novagen |
| Selection phage | Phage that eliminates a major class of λDE3 host range mutants | Novagen |
| T7 Tester phage | T7 RNA polymerase deletion mutant used in verifying lysogens in presence of IPTG | Novagen |

**Table S3.** Oligonucleotides used in this study.

| Name | Sequence (5’ - 3’) ^a^ | Description | Protein/recombinant protein | |
| --- | --- | --- | --- | --- |
| PaePBP4-A | 5’-CCCCATATGTTCAAGTCGCTGCGTACTC-3’ | Forward *dacB*-*Nde*I | PBP4HNC - PBP4HN |  |
| PaePBP4-B | 5’-TTCAAGCTTTTTCCGCGCGTGCAGGCTC-3’ | Reverse *dacB*-*Hind*III | PBP4HNC - PBP4HC - PBP4 |  |
| PaePBP4-C | 5’-CCCCATGTTCAAGTCGCTGCGTACTCTTG-3’ | Forward *dacB*-NcoI | PBP4HC |  |
| PaePBP4-D | 5’-CTCAAGCTTTTATTTCCGCGCGTGCAGG-3’ | Reverse *dacB*-*Hind*IIISC ^b^ | PBP4HN |  |
| PaePBP4-H | 5’-TTCGAATTCGATGTTCAAGTCGCTGCGT-3’ | Forward *dacB*-*Eco*RI | PBP4 |  |
| pUC/M13Fw | 5’-CCCAGTCACGACGTTGTAAAACG-3’ | Forward M13 | - |  |
| pUC/M13Rv | 5’-AGCGGATAACAATTTCACACAGG-3’ | Reverse M13 | - |  |
| T7 Promoter | 5’-TAATACGACTCACTATAGGG -3’ | Forward T7 | - |  |
| T7 Terminator | 5’-GCTAGTTATTGCTCAGCGG-3’ | Reverse T7 | - |  |
| pHERD/M13Fw | 5’-GTCACGACGTTGTAAAACGACGGCCAG-3’ | Forward pHERD26T | - |  |

*^a^* Sites for restriction endonucleases are underlined

*^b^* SC: TAA stop codon (green underline)
