# Supplementary material for: In vivo functional and molecular characterization of the Penicillin-Binding Protein 4 (DacB) of Pseudomonas aeruginosa
Source: BMC Microbiol. 2016 Oct 6;16:234. doi: 10.1186/s12866-016-0853-x (PMC5054556; doi:10.1186/s12866-016-0853-x)
Supplement: Additional file 2: — Table S1. HPLC analysis of muropeptides prepared from the peptidoglycan of Pseudomonas aeruginosa UCBPP-PA14 grown under natural conditions, with overexpression of LMM-PBP4 and antibiotic inactivation. Table S2. Kinetic parameters (V max, K m, k cat) for LMM-PBP4 of Pseudomonas aeruginosa O1 on natural substrates M5 and D45. (DOCX 29 kb) [file 12866_2016_853_MOESM2_ESM.docx]

**Table S1.** HPLC analysis of muropeptides prepared from the peptidoglycan of *Pseudomonas aeruginosa* UCBPP-PA14 grown under natural conditions, with overexpression of LMM-PBP4 and antibiotic inactivation.

**Relative abundance (mol%) *^a^***

| Strain | Monomers | Dimers | Trimers | DAP-DAP | Lpp | Anhydrous forms | Pentapeptide | Cross-linkage (%) | D-D/total (%) | Chain length |
| --- | --- | --- | --- | --- | --- | --- | --- | --- | --- | --- |
| PA14WT | 63.9 | 32.6 | 2.4 | 1.1 | 1.6 | 8.1 | 0.4 | 37.6 | 3.0 | 12.2 |
| PA14WT/pHERD-PBP4 uninduced | 67.5 | 29.4 | 1.9 | 1.0 | 1.9 | 7.3 | 0.4 | 33.4 | 2.8 | 13.6 |
| PA14WT/pHERD-PBP4 + Arabinosa 0.2% | 76.8 | 21.9 | 1.2 | 1.1 | 1.9 | 4.4 | 0.3 | 24.3 | 3.5 | 22.2 |
| PA14WT + FOX | 59.1 | 37.3 | 3.4 | 0.9 | 1.2 | 8.6 | 3.7 | 44.5 | 2.0 | 11.6 |
| PA14WT/pHERD-PBP4 + Arabinosa 0.2% + FOX | 65.8 | 31.7 | 2.3 | 1.1 | 1.7 | 8.1 | 2.1 | 36.6 | 3.1 | 12.2 |

D-D/total is the ratio of DAP-DAP cross-links to the

total cross-links. The averages of data from three measurements from biological replicates are shown

D-D/total is the ratio of DAP-DAP cross-links to the

total cross-links. The averages of data from three measurements from biological replicates are shown

D-D/total is the ratio of DAP-DAP cross-links to the

total cross-links. The averages of data from three measurements from biological replicates are shown. S

*^a^* Relative abundance in mole % of different types of muropeptides: DAP-DAP, muropeptides having DAP-DAP peptide bridges; Lpp, muropeptides bound to C-terminal Arg-Lys dipeptide of Braun’s lipoprotein; anhydrous form, muropeptide contains a 1,6-anhydroMurNAc residue; pentapeptide, muropeptides having pentapeptide stem; cross-linkage, degree of peptidoglycan crosslinking in percentage; D-D/total, percent ratio of DAP-DAP crosslinks to total peptidoglycan crosslinks; chain-length, peptidoglycan length. Values are means from three independent experiments.

**Table S2.** Kinetic parameters (*V_max_*, *K_m_*, *k_cat_*) for LMM-PBP4 of *Pseudomonas aeruginosa* O1 on natural substrates M5 and D45 *^a^*.

**Substrate M5**

| **Enzyme** | ***V_max_*** (μmol M4·min^-1^·μg PBP4^-1^) | ***K_m_*** (μM) | ***k_cat_*** (s^-1^) |  |
| --- | --- | --- | --- | --- |
| **LMM-PBP4** | 7.7 ± 1.3 x 10^-5^ | 71.9 ± 2.1 | 1.1 ± 0.2 x 10^-4^ |  |

**Substrate D45**

| **Enzyme** | ***V_max_*** (μmol M4·min^-1^·μg PBP4^-1^) | ***K_m_*** (μM) | ***k_cat_*** (s^-1^) |  |
| --- | --- | --- | --- | --- |
| **LMM-PBP4** | 6.2 ± 0.7 x 10^-4^ | 32.8 ± 3.1 | 2.1 ± 0.4 x 10^-3^ |  |

**Substrate D45**

| **Enzyme** | ***V_max_*** (μmol M5·min^-1^·μg PBP4^-1^) | ***K_m_*** (μM) | ***k_cat_*** (s^-1^) |  |
| --- | --- | --- | --- | --- |
| **LMM-PBP4** | 5.5 ± 1.1 x 10^-4^ | 33.4 ± 1.8 | 1.8 ± 0.1 x 10^-3^ |  |

*^a^* All kinetic constants must be considered apparent values because of the impossibility of calculating initial enzyme velocities by HPLC. The results correspond to the mean value ± standard deviations of experiments done in triplicate. Data for substrate M5 were calculated from the analysis of the M4 reaction product, and data for substrate D45 were calculated using both M4 and M5 reaction products.
